# Electromyographic Patterns during Golf Swing: Activation Sequence Profiling and Prediction of Shot Effectiveness

**DOI:** 10.3390/s16040592

**Published:** 2016-04-23

**Authors:** Antanas Verikas, Evaldas Vaiciukynas, Adas Gelzinis, James Parker, M. Charlotte Olsson

**Affiliations:** 1 Intelligent Systems Laboratory, Centre for Applied Intelligent Systems Research, Halmstad University, Kristian IV:s väg 3, PO Box 823, Halmstad S-30118, Sweden; antanas.verikas@hh.se; 2 Department of Electrical Power Systems, Kaunas University of Technology, Studentu 50, Kaunas LT-51368, Lithuania; adas.gelzinis@ktu.edu; 3 Department of Information Systems, Kaunas University of Technology, Studentu 50, Kaunas LT-51368, Lithuania; 4 School of Business, Engineering and Science, Halmstad University, Kristian IV:s väg 3, PO Box 823, Halmstad S-30118, Sweden; james.parker@hh.se (J.P.); charlotte.olsson@hh.se (M.C.O.)

**Keywords:** EMG, muscle activity onset, peak detection, random forest, decision fusion

## Abstract

This study analyzes muscle activity, recorded in an eight-channel electromyographic (EMG) signal stream, during the golf swing using a 7-iron club and exploits information extracted from EMG dynamics to predict the success of the resulting shot. Muscles of the arm and shoulder on both the left and right sides, namely *flexor carpi radialis*, *extensor digitorum communis*, *rhomboideus* and *trapezius*, are considered for 15 golf players (∼5 shots each). The method using Gaussian filtering is outlined for EMG onset time estimation in each channel and activation sequence profiling. Shots of each player revealed a persistent pattern of muscle activation. Profiles were plotted and insights with respect to player effectiveness were provided. Inspection of EMG dynamics revealed a pair of highest peaks in each channel as the hallmark of golf swing, and a custom application of peak detection for automatic extraction of swing segment was introduced. Various EMG features, encompassing 22 feature sets, were constructed. Feature sets were used individually and also in decision-level fusion for the prediction of shot effectiveness. The prediction of the target attribute, such as club head speed or ball carry distance, was investigated using random forest as the learner in detection and regression tasks. Detection evaluates the personal effectiveness of a shot with respect to the player-specific average, whereas regression estimates the value of target attribute, using EMG features as predictors. Fusion after decision optimization provided the best results: the equal error rate in detection was 24.3% for the speed and 31.7% for the distance; the mean absolute percentage error in regression was 3.2% for the speed and 6.4% for the distance. Proposed EMG feature sets were found to be useful, especially when used in combination. Rankings of feature sets indicated statistics for muscle activity in both the left and right body sides, correlation-based analysis of EMG dynamics and features derived from the properties of two highest peaks as important predictors of personal shot effectiveness. Activation sequence profiles helped in analyzing muscle orchestration during golf shot, exposing a specific avalanche pattern, but data from more players are needed for stronger conclusions. Results demonstrate that information arising from an EMG signal stream is useful for predicting golf shot success, in terms of club head speed and ball carry distance, with acceptable accuracy. Surface EMG data, collected with a goal to automatically evaluate golf player’s performance, enables wearable computing in the field of ambient intelligence and has potential to enhance exercising of a long carry distance drive.

## 1. Introduction

The capability of hitting long distances in golf always had a lot of attention in both the research and coaching literature. Recent studies highlight the importance of driving distance in relation to golf performance [1], and the strategic advantage gained by hitting irons further has been theorized to give advantageous ball trajectory conditions, which increase the possibility of higher accuracy [2]. Identifying factors that influence the ability to hit further with the irons is an important area for developing golf performance. Initial ball velocity is dependent on the centeredness of impact, club head velocity (*i.e.*, magnitude and direction) and club face orientation [3,4]. Understanding the muscle activation patterns that characterize an effective golf swing has potential to facilitate golf coaching [5], as well as injury prevention [6].

Surface electromyography (EMG) data-based predictive models have been applied in a variety of fields. Prostheses control by mapping EMG signals into several classes of forearm motions [7,8,9,10,11,12,13,14,15,16,17,18,19], evaluation of local muscle fatigue in static [20,21,22,23,24], as well as dynamic exercises [25,26,27,28,29], screening for neuromuscular disorders [30,31,32,33] and analysis of the gait of arthritic patients [34,35] are probably the main application fields. Prediction of externally-applied forces to human hands by relating the EMG signals and experimentally known forces [36], prediction of forces on the lumbar spine [37], estimation of the iso-kinetic knee torque [38], recognition of simple and complex finger flexions [39], anticipation of head motion [40] and finger joint angle [41] for virtual-environment applications, distinguishing between normal subjects and subjects that experienced stroke of several degrees of severity (severe, moderate and mild) [42], measuring age-related reduction in number of motor units [43], a computer interface for text typing based on EMG signals induced by wrist movements [44], hand gesture sensing [45], assessing the subject’s vigilance level [46], stride detection and stride length estimation during walking [42] and classification of different tremor types [47] are other recent examples of using EMG data-based predictive models.

While predictive modeling using EMG features as predictors is a popular topic in various EMG signal analysis-based applications, prediction of shot success from EMG activity during golf swing is rarely researched. All EMG studies relevant to the golf swing were synthesized in [48], enabling the explanation of why certain muscles are active during specific phases of the swing in relation to the movement of the body during the swing. A recent review in [49] found 19 articles regarding EMG variables of the golf swing, but none of the reviewed articles is dedicated to studying relations between EMG signals and data characterizing biomechanical swing kinematics and/or the effectiveness parameters of the resulting shot. A comparison of two different threshold methods for EMG activity onset timing during the golf swing [50] found that club type has no influence on onset detection, and there is a similar activation time between muscles on the right and left body sides.

Based on the swing kinematics, some studies [51,52] find that neither upper torso nor pelvis rotation alone, but rather the X-factor (upper torso and pelvis separation) and delayed initiation of movement of the arms and wrists in the downswing are significantly and strongly correlated with ball velocity. A biomechanical analysis of professional swings in [53] also concludes that the effectiveness of the swing for a long drive appears to be related to a sequential activation of muscle groups that optimizes transfer of momentum through the kinetic chain, and high club velocity can be achieved. It is widely agreed that a biomechanically-correct transition, as far as the golf swing progression is concerned, works up from the hips (pelvis) to the chest (thorax), then the lead arm and, finally, the club.

Noting the lack of research on EMG temporal patterns generated during a golf swing and the potential of using information on EMG dynamics for the prediction of shot success, we try to address these aspects in our study. Predictive models are constructed here to predict club head speed or ball carry distance from EMG features. Data from golf players are described in Section 2. Proposed and employed methods are outlined in Section 3. The performed experiments are summarized in Section 4. Results are reviewed in Section 5, and final remarks are made in Section 6.

## 2. Equipment and Data

Fifteen elite golf players, 10 men and five women (mean age = 22 ± 2 years), with a handicap of −1.5 or better, were considered for the study. All participants were right-handed. Each subject was instructed to take five “normal” swings with their own 7-iron and to hit into a net, eight meters from the tee area. The total swing time ranged between 1.5 and 2.2 s.

**Figure 1:**
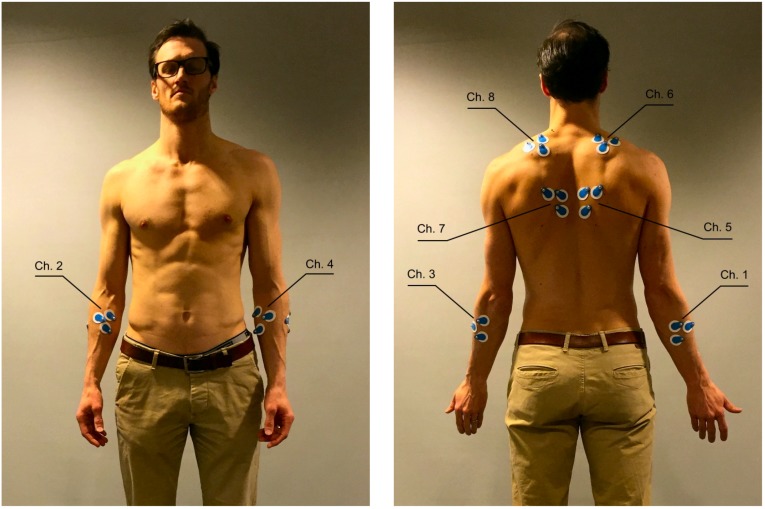
Participant body with eight-channel EMG recorder electrodes attached. Electrodes on *extensor digitorum communis (EDC)* arm muscles can be seen in the front side view (**Left**); electrodes on *flexor carpi radialis (FCR)* arm muscles, as well as electrodes on *rhomboideus* and *trapezius* muscles can be seen in the back side view (**Right**). Mapping between a channel number and a muscle: (1) right *FCR*; (2) right *EDC*; (3) left *FCR*; (4) left *EDC*; (5) right *rhomboideus*; (6) right *trapezius*; (7) left *rhomboideus*; and (8) left *trapezius*.

Surface EMG signals were collected with bipolar surface electrodes collected by an eight-channel EMG recorder (ME Muscle Tester 6000 Megawin Electronics, Kuopio, Finland), recorded at a 1000-Hz sampling frequency. Electrodes were placed with a 20-mm inter-electrode distance along the muscle belly of each muscle, with a reference electrode placed perpendicular to the measurement electrodes to minimize EMG cross-talk. The muscles investigated were the left and right hands’ *extensor digitorum communis (EDC)*, *flexor carpi radialis (FCR)* and the left and right side of *rhomboideus* and *trapezius*. Exact locations and mapping of sensors on the body are illustrated in Figure 1. More details on the placement of sensors are in Table A1.

**Figure 2:**
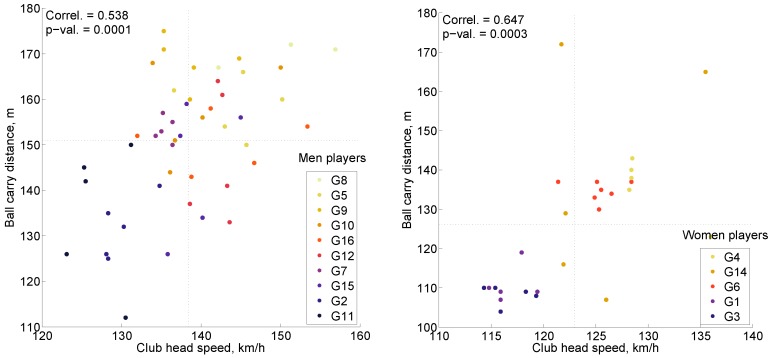
Scatterplot, showing the effectiveness for men (**Left**) and women (**Right**) players. Notes: grid lines correspond to sex-specific averages; shots are colored according to the player’s overall effectiveness, where a lighter color means a more effective player, based on average club head speed and average ball carry distance through all of his/her shots.

The Flightscope X2 Doppler-radar launch monitor system (Flightscope, Version 6.0.9, Stellenbosch, South Africa) measured ball carry distance, lateral distance (left/right), club and ball speed, swing height, flight time, horizontal and vertical launch angles and vertical impact angle. Club head speed and ball carry distance parameters were selected as measures of shot effectiveness. Figure 2 shows the effectiveness of each shot with respect to these parameters. Pearson’s correlation coefficient between speed and distance was statistically significant at 99% confidence (*p*-value < 0.01) and positive, which conforms to previous research [54]. Shots of men had moderate (0.538) and shots of women had strong (0.647) positive correlation. Men had a noticeably higher sex-specific average for both parameters. The most effective player was G8 for men and G4 for women, whereas the least effective player was G11 for men and G3 for women.

## 3. Methodology

The most popular techniques used to extract features characterizing EMG signals are autoregressive modeling, higher order frequency moments, multi-resolution wavelet analysis, the maximum eigenvalues of the time-varying covariance patterns between EMG channels, signal variance values computed in overlapping windows, techniques based on fractal analysis, principle component analysis, independent component analysis, non-negative matrix factorization, short time Fourier transform and the orthogonal fuzzy neighborhood discriminant analysis.

We use time domain features in this work, assuming that such features are better at describing the underlying muscle orchestration than frequency domain features. By muscle activation pattern, we refer to the orchestration of EMG activity onsets in eight muscles of the arm and shoulder, namely right and left sides for *extensor digitorum communis (EDC)*, *flexor carpi radialis (FCR)*, *rhomboideus* and *trapezius*, during a golf swing. A comparison of onset sequences through EMG channels between swings of the same player and between different players is done visually.

Channel-specific statistics of filtered EMG signal, pairwise correlations between filtered EMG dynamics in various muscles during swing, the details of EMG signal peaks in each muscle and the concurrency of peak positions are computed as predictive features for the random forest model. Using computed features, the regression task seeks to estimate the target attribute value (club head speed or ball carry distance), and the classification task seeks to detect an effective shot, where effectiveness corresponds to being above the personal average of the player in question.

### 3.1. Muscle Activation Sequence Profiling

**Figure 3:**
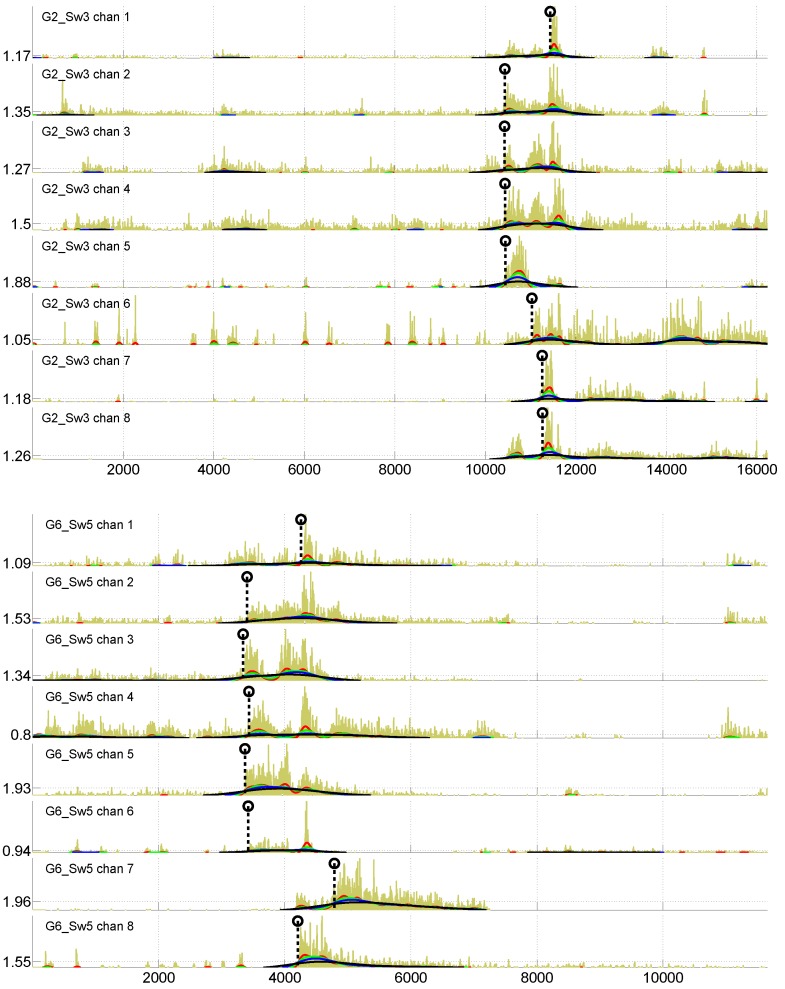
EMG signals recorded from eight muscles of golf players G2 (**Top**) and G6 (**Bottom**). Note: position in samples on the *X* (horizontal) axis. Channel numbers correspond to the following muscles: (1) right *FCR*; (2) right *EDC*; (3) left *FCR*; (4) left *EDC*; (5) right *rhomboideus*; (6) right *trapezius*; (7) left *rhomboideus*; and (8) left *trapezius*.

We expect to obtain some insights into golf swing patterns by studying muscle activation profiles, characterized by EMG signals recorded from different muscles. Figure 3 present two examples of EMG signals recorded from two golf players. On the *X* axis is shown a sample number (1000 samples/s is the sampling frequency), while the *Y* axis reflects the absolute value of an EMG signal amplitude. Since noise is always present in EMG signals, some preprocessing of EMG signals is needed to obtain robust characterization of the muscle activation sequence. Therefore, in addition to the raw EMG signal, information resulting from signal preprocessing is also shown in Figure 3.

We define a muscle activation profile as a vector, x, of time differences between the start of swing-related activation in the reference muscle and the other seven muscles. Thus, x consists of seven components $x=x_{1},...,x_{7}$. Channel 8 (left *trapezius*) was used as the reference for activation times in this work. The start of swing-related activations, marked by a dashed vertical line ending with a circle in Figure 3, are to be determined in all eight channels, to obtain components of x. This is not an easy task, since rather substantial activation can be observed in some muscles, even before the golf swing start (see Figure 3). To detect the beginning of swing-related activations in different channels, the following processing steps are performed.

1. Filter the signal |s(t)| in each channel *i* by four sliding Gaussian filters $G_{1}$, $G_{2}$, $G_{3}$ and $G_{4}$ of the following width: 256, 512, 1024, 2048 samples. The resulting signals $v_{1}^{i}\left(t\right),...,v_{4}^{i}\left(t\right)$ are shown in Figure 3 by the red, green, blue and black curves.
2. For each channel *i*, determine a threshold value $T^{i}$:
  (1) $$T^{i}=\frac{\alpha }{card\{\sum \}}\sum_{t=t_{v_{4max}^{i}}-\delta }^{t=t_{v_{4max}^{i}}+2\delta }\left|s^{i}\left(t\right)\right|,\;\;i=1,...,8$$
  where card{∑} means the number of elements in the sum, *α* and *δ* are parameters selected experimentally and $t_{v_{4max}^{i}}$ stands for the position of the maximum value of the *i*-th channel signal filtered by the $G_{4}$ filter. Values of α=1.2 and δ=500 worked well in our study. The determined thresholds are shown on the *Y* axis in Figure 3.
3. The start of swing-related activation in the *i*-th channel $t_{s}^{i}$ is then given by:
  (2) $$t_{s}^{i}=\arg\max_{t=min_{t}(|t-t_{T^{i}}^{v_{2,3,4}}\left|\right)}\left\{\frac{\partial v_{1}^{i}\left(t\right)}{\partial t}\right\}$$
  where $t_{T^{i}}^{v_{2,3,4}}$ stands for three time moments, where $v_{2}^{i}\left(t\right)=T^{i}$, $v_{3}^{i}\left(t\right)=T^{i}$ and $v_{4}^{i}\left(t\right)=T^{i}$.
4. Define *λ* as the maximum difference in activation time between the reference channel and all remaining channels:
  (3) $$\lambda =max_{i=1,...,7}\left(\right|t_{s}^{8}-t_{s}^{i}\left|\right)$$
  Inspect and post-process the results manually if $\lambda <\lambda_{min}$ or $\lambda >\lambda_{max}$, where global $\lambda_{min}$ and $\lambda_{max}$ are determined experimentally from all EMG channels and swings of all participants.

The determined $t_{s}^{i}$ values constitute components $x_{i}$ and x. In Figure 3, the determined $t_{s}^{i}$ values are shown by dashed vertical lines with a circle on the top.

### 3.2. EMG Features for Predictive Modeling

**Figure 4:**
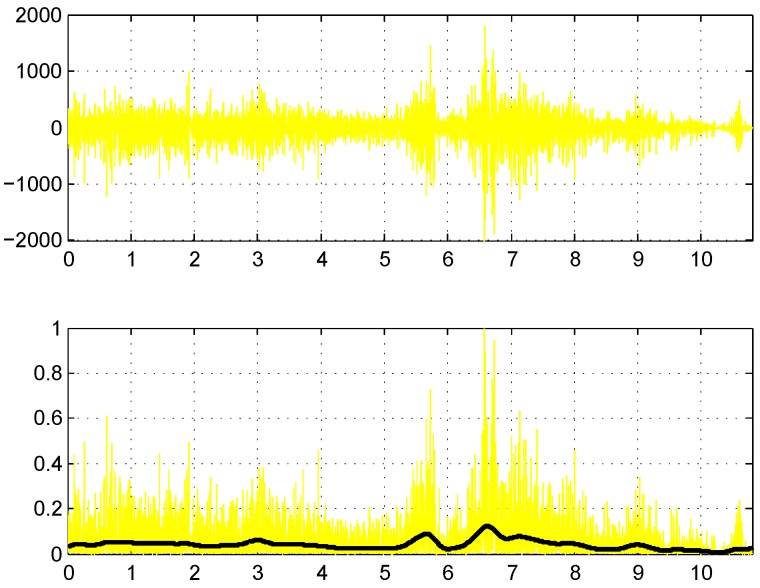
EMG signal pre-processing: raw signal (**Top**) and normalized rectified signal (**Bottom**) with the result of Butterworth filtering (black curve). Note: the position is in seconds on the *X* (horizontal) axis.

Pre-processing of an EMG signal usually corresponds to full-wave rectification, followed by filtering of the rectified signal to obtain a smooth linear envelope of the EMG. Most software packages for EMG analysis in sports biomechanics use a fourth-order Butterworth filter [55]. We apply a second-order Butterworth filter for both high- and low-pass filtering. To eliminate phase lag, resulting from a second-order filter, the signal is filtered again in the reverse direction, which effectively converts the filter to a fourth-order filter. Low-pass filtering follows after demeaning the high-pass filtering result, and the cut-off frequency is 20 and 400 Hz for high- and low-pass, respectively. An example of the filtered signal is provided in the bottom part of Figure 4. The Butterworth high-pass filter with a corner frequency of 20 Hz is also recommended by [56] for general use.

**Figure 5:**
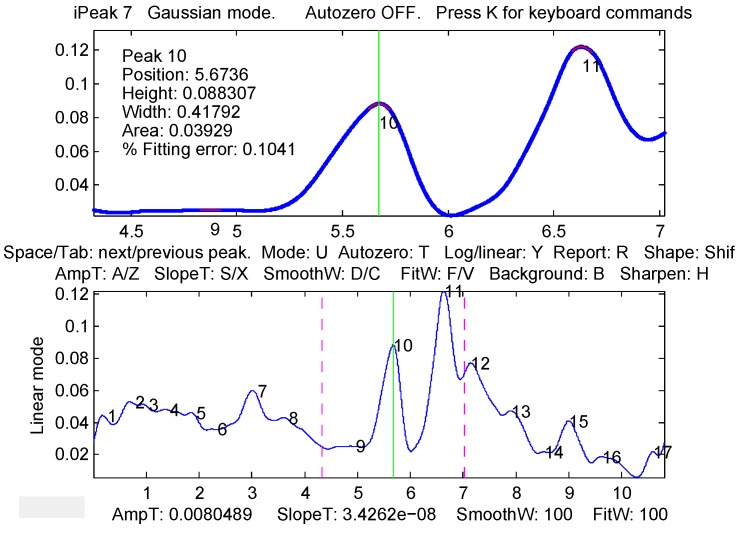
Finding peaks in the filtered EMG signal. Interface window of the *ipeak* function: entire pre-processed signal (**Bottom**) with 17 peaks detected and the zoomed-in portion of the signal (**Top**) containing the two highest peaks (# 10 and # 11). Note: the position is in seconds on the *X* (horizontal) axis.

Peak detection, which is at the core of feature extraction here, is done using a MATLAB function *ipeak* from the package *ipeak7.zip* (Version 7.1), created and maintained by Prof. Thomas C. O’Haver [57]. A program window with the *ipeak* function result for the filtered EMG signal from a single channel is displayed in Figure 5. To find peaks and extract a swing segment from the surface EMG signal, the following steps are performed.

1. Normalize the rectified signal |s(t)| in each channel *i* to the peak activation level (see Figure 4):
  (4) $$normalize\left(s\right)=\frac{\left|s(t)\right|}{\max(\left|s(t)\right|)}$$
2. Filter the normalized signal of each channel *i* using the high- and low-pass Butterworth filters (the result can be seen as the resulting black curve in the bottom part of Figure 4).
3. In each channel *i*, detect all peaks in the filtered signal using the *ipeak* function (with maximum peak density parameter PeakD = 2), and retain the two highest peaks, denoting them in the order of appearance as $P_{1}^{i}$ and $P_{2}^{i}$. In Figure 5, Peak # 10 would correspond to $P_{1}^{i}$, and Peak # 11 would correspond to $P_{2}^{i}$.
4. Pool absolute time-based positions of retained peaks from all channels into a single vector of positions p. Enumerate all possible *i* values into two identical lists ${\mathrm{idx}}_{1}$ and ${\mathrm{idx}}_{2}$ of channel indexes.
5. Check for outliers among the elements of the vector p using the box-plot rule, finding values exceeding the 1.5 inter-quartile range above/below the upper/lower quartile. If outliers are found, remove the numbers of the corresponding channels from ${\mathrm{idx}}_{1}$ and/or ${\mathrm{idx}}_{2}$.
6. Compute swing start ($t_{1}$) and end ($t_{2}$) times by using the position and width of the marginal $P_{1}$ and $P_{2}$ peaks:
  (5) $$k=\arg\min_{i\in {\mathrm{idx}}_{1}}\left(pos\left(P_{1}^{i}\right)\right)\;\mathrm{and}\;t_{1}=pos\left(P_{1}^{k}\right)-\frac{width(P_{1}^{k})}{2}$$
  (6) $$k=\arg\max_{i\in {\mathrm{idx}}_{2}}\left(pos\left(P_{2}^{i}\right)\right)\;\mathrm{and}\;t_{2}=pos\left(P_{2}^{k}\right)+\frac{width(P_{2}^{k})}{2}$$
  where *k* is the channel number of the marginal peak and functions pos and width return the position and width of the peak.
7. Retain only the segment from $t_{1}$ to $t_{2}$.
8. If outliers were found (in Step # 5), proceed from Step # 3.
9. Convert an absolute position of each peak within the signal into a relative position within the swing by taking into account the swing duration ($t_{2}-t_{1}$).

After extracting the swing segment, the feature vector x is determined by the feature set, where the very first feature $x_{0}$ is always sex (0, woman; 1, man), and the remaining features are related to: (1) channel statistics and correlations; (2) properties of the first peak; (3) properties of the second peak; (4) properties derived from both peaks.

Table 1 presents a list of feature sets used in this work. Statistics, used to enrich Feature Sets # 1, 2, 5, 6, 8, 9, 11, 12, 15–22 with various aspects of the distribution in question, encompass the following 12 characteristics: minimum, maximum, mean, median, lower quartile ($Q_{lo}$), upper quartile ($Q_{up}$), standard deviation, inter-quartile range, lower range (median, $Q_{lo}$), upper range ($Q_{up}$, median), skewness and kurtosis. Since there are 4 channels on one side and the EMG dynamics of each channel is compressed into 12 characteristics, Feature Sets # 1, 2 (*ChanStats*) contain 48 (4 × 12) features plus an extra feature for the duration in seconds; therefore, 49 features in total.

The main properties of a peak, estimated using the *ipeak* function (see the top part of Figure 5), are the following: position, height, width, area and fitting error. All peak properties are used in Feature Sets # 7, 10, 13, 14, while Feature Sets # 8, 9, 11, 12, 15–22 are calculated using peak position only. Feature Sets # 13, 14 are based on the mathematical operation between properties of the first and second peaks, where Feature Set # 13 uses subtraction and Feature Set # 14 uses the division of the corresponding properties. Features Sets # 7, 10, 13, 14 are constructed considering 5 properties for each of the 8 channels; therefore, they contain 40 (5 × 8) features in total.

Feature Sets # 3–6, 8, 9, 11, 12, 15–22 are derived from all possible channel pairs. Having 8 channels, we obtain 28 arrangements. Correlation-related feature sets are based on the arithmetical operation between Spearman and Pearson correlations for each pair of channels. Feature Sets # 3, 5 (containing *CorrDelta*in their name) consider subtraction, and Feature Sets # 4, 6 (containing *CorrRatio*in their name) consider the division of correlations for the corresponding channel pair.

Peak position-based Feature Sets # 8, 9, 11, 12, 15–22 (containing *Sync* in their name) evaluate peak position concurrency for each pair of channels. The concurrency is measured here as a signed difference for Feature Sets # 8, 11, 15–18 or an absolute difference for Feature Sets # 9, 12, 19–22 (containing *Abs*in their name). Feature Sets # 8, 9, 11, 12 are constructed using the difference between positions of the 1st and 2nd peaks separately. These separate concurrences are further combined by the basic arithmetical operations: subtraction in Feature Sets # 15, 19; addition in Feature Sets # 16, 20; multiplication in Feature Sets # 16, 21; division in Feature Sets # 17, 22.

### 3.3. Prediction Using Random Forest

Regression models seek to predict club head speed or the ball carry distance of a shot. Binary classification models try to detect the personal effectiveness of speed or distance, that a resulting shot for a player in question is *effective* or *ineffective* in terms of speed or distance. Regression has a continuous, while detection has a discreet target attribute. Discretization into labels for the detection task is done with respect to the player-specific average: a shot resulting in being above the personal average club head speed or ball carry distance is considered as *effective*, and a shot resulting in being below the personal average club head speed or ball carry distance is considered as *ineffective*.

**Figure 6:**
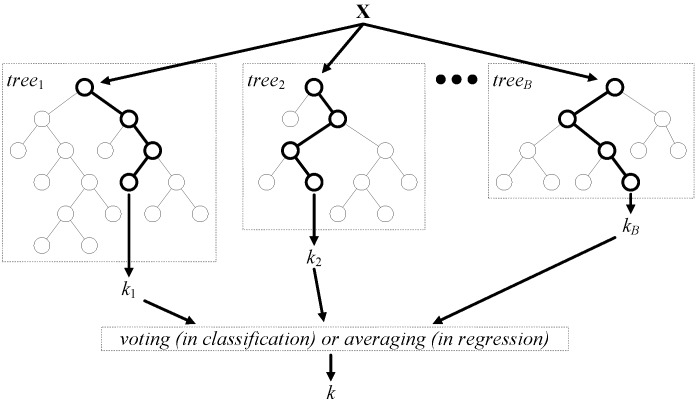
Architecture of the random forest model.

We used random forest (RF) [58] for predictive modeling. The MATLAB port of the original RF algorithm was obtained from [59]. RF can be used for both classification and regression. In classification, RF is a committee of decision trees, where the final output is derived from voting (see Figure 6). In regression, voting by a committee is replaced with averaging. Random forests have been successfully applied in a variety of fields [60]. The core idea of RF is to combine many (*B* in total) decision trees, built using different bootstrap samples of the original dataset and a random subset (of predetermined size *q*) of features $x^{1},\ldots ,x^{p}$ (see Figure 6). RF is known to be robust against over-fitting, and as the number of trees increases, the generalization error converges to a limit [58]. Low bias and low correlation are essential for the robust generalization performance of the ensemble. To get low bias, trees are unpruned (grown to the maximum depth). To achieve the low correlation of trees, randomization is applied. RF is constructed in the following steps:

1. Choose the forest size *B* as a number of trees to grow and the subspace size q≤p as a number of features to provide for each tree node.
2. Draw a bootstrap sample (random sample with replacement) of the learning dataset, which generally results in $∼\frac{2}{3}\cdot n$ unique observations for training, thus leaving $∼\frac{1}{3}\cdot n$ for testing as the out-of-bag (OOB) dataset for that particular tree.
3. Grow an unpruned tree using the bootstrap sample. When growing a tree, at each node, *q* variables are randomly selected out of the *p* available.
4. Repeat Steps 2 and 3 until the size of the forest reaches *B*.

Because of the “repeated measures” aspect in golf data, where each player is represented by several swings, sampling part of the RF had to be modified to ensure that all swings of each player are either included in a bootstrap sample or left aside as OOB. The list of passed parameters to the external interface function (C++ source code to build MATLAB mex files) was expanded by the additional indexList parameter, containing subject’s unique ID for each swing. The implemented modification of bootstrap sampling converts leave-one-out validation into leave-one-subject-out validation. Furthermore, the RF setting, allowing to perform stratified sampling, was configured in the classification task to preserve the sex ratio of the full dataset in each drawn bootstrap sample.

### 3.4. Decision-Level Fusion

Individual RFs were built independently on bootstrap sets, and the decisions of these individual experts were combined in a meta-learner fashion. RF was used in this work both as a base learner and as a meta learner. This implies that outputs from RF models from the first stage are treated as inputs (meta-features) for another RF in the second stage. The set of meta-features was slightly expanded by always including sex and intentionally avoiding removal of sex in the decision optimization stage.

For the classification task, an input to the meta-learner is the difference between class *a posteriori* probabilities obtained from the base-learner. Given a trained RF, this difference is estimated as:

(7) $$d\left(\left\{t_{1},...,t_{L}\right\},x\right)=\frac{\sum_{i=1}^{L}f\left(t_{i},x,q=2\right)}{L}-\frac{\sum_{i=1}^{L}f\left(t_{i},x,q=1\right)}{L}$$

where x is the object being classified, *L* is the number of trees $t_{1},...,t_{L}$ in the random forest for which observation x is OOB, *q* is a class label and $f(t_{i},x,q)$ stands for the *q*-th class frequency in the leaf node, into which x falls in the *i*-th tree $t_{i}$ of the forest:

(8) $$f\left(t_{i},x,q\right)=\frac{n(t_{i},x,q)}{\sum_{j=1}^{Q}n\left(t_{i},x,q_{j}\right)}$$

where *Q* is the number of classes and $n(t_{i},x,q)$ is the number of training data from class *q* and falling into the same leaf node of $t_{i}$ as x.

For the regression task, input to the meta-learner is the averaged OOB prediction of the base-learner. Given a trained RF, this estimate is provided by:

(9) $$\begin{array}{cccc} & d(\left\{t_{1},...,t_{L}\right\},x) & = & \frac{\sum_{i=1}^{L}f\left(t_{i},x\right)}{L} \end{array}$$

where x is the data instance represented by the feature set in question, *L* is the number of trees $t_{1},...,t_{L}$ in the random forest for which the observation x is OOB and $f(t_{i},x)$ corresponds to the predicted value of target attribute.

Meta-features were also investigated by performing permutation-based variable importance analysis. The mean decrease in accuracy (for the detection task) or the mean decrease in root mean squared error (for the regression task) were considered here as variable importance measures. Each meta-feature is processed by permuting its values several times, and the mean difference in RF performance on OOB data is estimated.

### 3.5. Decision Optimization

In ensemble learning, non-generative ensemble or decision optimization [61,62] refers to model selection through tuning of the meta-learner and/or selection of meta-features. RF parameter *B* was fixed throughout experiments, but parameter *q* was selected in a grid search (for individual RFs) or brute force (for combiner RF) fashion. All features were used for individual RFs. Decision optimization to search out a subset of meta-features was done only for the RF combiner through a specific variant of the evolutionary algorithm. Each generated meta-feature subset was evaluated with all possible *q* values, and the best evaluation, summarized using the cost of log-likelihood ratio (C_llr_, see Section 3.6.1 for details), is returned as a result of the fitness function.

We have chosen angle-modulated differential evolution (AMDE) as the decision optimization technique for wrapper-based meta-feature selection in the meta-learner model. AMDE was initially proposed in [63] as a homomorphous mapping technique enabling the differential evolution algorithm to operate within a binary space. In [64], AMDE was used to select features for support vector machine, while in [65], AMDE was applied for decision optimization of meta-learner RF.

The angle modulation (AM) technique uses a generating function to transform a 4-dimensional continuous-valued space into a larger binary-valued *n*-dimensional Hamming space (*n* is the total number of individual feature sets), which corresponds to convenient tuning of a feature inclusion mask by optimizing 4 continuous parameters. The transformation is achieved using the following trigonometric function:

(10) g(x)=sin(2π(x-a)×b×cos(2π×c(x-a)))+d

where the coefficients *a*, *b*, *c* and *d* are four optimized parameters determining the shape of the generating function (*a*, horizontal shift; *b*, maximal sin frequency; *c*, maximal cos frequency; *d*, vertical shift) and *x* is an element from a set (size *n*) of evenly-spaced values, for example, starting from 0.1 with a step of 0.1 until 0.1*n is reached. We generate a binary selection vector g by:

(11) $$g_{k}=\left\{\begin{array}{cc} 1 & \mathrm{if}\;g(x_{k})>0\\ 0 & \mathrm{if}\;g(x_{k})\leq 0 \end{array}\right.$$

where k=1⋯p and the $g(x_{k})$ value is calculated using Equation (10). By proceeding with *k*, a vector g of length *n* is generated. The vector g represents the potential solution within the binary problem space; in our case, a binary mask of the meta-features to select.

### 3.6. Evaluating Predictive Performance

Generalization performance of RF was evaluated by OOB validation. It is well known that for large RF, each observation of a learning set is used as an OOB observation, and the OOB performance estimate corresponds to an unbiased estimate of a test set error.

#### 3.6.1. Assessing Detection

To evaluate the goodness of detection, detector’s scores for OOB data were obtained. Votes of RF were converted to a proper score vector by normalizing votes for a specific class through division by the total number of times the case was OOB. Using soft decisions (scores), instead of hard decisions, enables more precise evaluation of the detection. We evaluated scores from RF using several measures: (1) the cost of the log-likelihood ratio (C_llr_); (2) the detection error trade-off (DET) curve and the equal error rate (EER); (3) the receiver operating characteristic (ROC) curve and the area under the curve (AUC).

Various thresholds are possible to convert a soft decision into hard, resulting in a multitude of confusion matrices, and the overall performance of the detector can be visually summarized by the ROC or DET curve. Due to the logarithmic scale, the DET curves allow a comparison of several systems at a glance more easily than the ROC curves [66]. A quick way to compare the accuracy of detectors with different DET (ROC) curves is the equilibrium point, widely known as the equal error rate (EER). EER is the point where the DET (ROC) curve intersects the diagonal and: (1) false positive rate (miss rate) = false negative rate (false alarm rate), for DET; or (2) true positive rate (sensitivity) = true negative rate (specificity), for ROC.

The cost of the log-likelihood-ratio (C_llr_) is the most comprehensive goodness-of-detection metric, used here as the main criterion for model selection and ranking of predictive models. The log-likelihood-ratio is the logarithm of the ratio between the likelihood that the shot was *effective* and the likelihood that the shot was *ineffective*. More details on C_llr_ are provided in [67]. The C_llr_, DET, EER, ROC and AUC measures were estimated using the ROC convex hull method, available in the BOSARIS toolkit [67]. In a detection task, a well-calibrated and useful detector should have C_llr_ < 1, EER < 50% and AUC > 0.5.

#### 3.6.2. Assessing Regression

Two types of errors were used: the root mean squared error (RMSE) and the mean absolute percentage error (MAPE). Pearson’s correlation coefficient between actual and predicted values was also computed. RMSE is used here as the main criterion for model selection and ranking of predictive models.

The reference values for RMSE, MAPE and correlation were estimated by considering a variant of naive prediction, which simply outputs a sex-specific average instead of a real attempt at prediction. Satisfactory performance, which is better than using a sex-specific average as prediction, can be confirmed when: (1) RMSE < 6.91, MAPE < 4.12 and correlation > 0.742 for club head speed; (2) RMSE < 13.37, MAPE < 8.15 and correlation > 0.738 for ball carry distance.

## 4. Experimental Investigations

### 4.1. Muscle Activation Profiles

**Figure 7:**
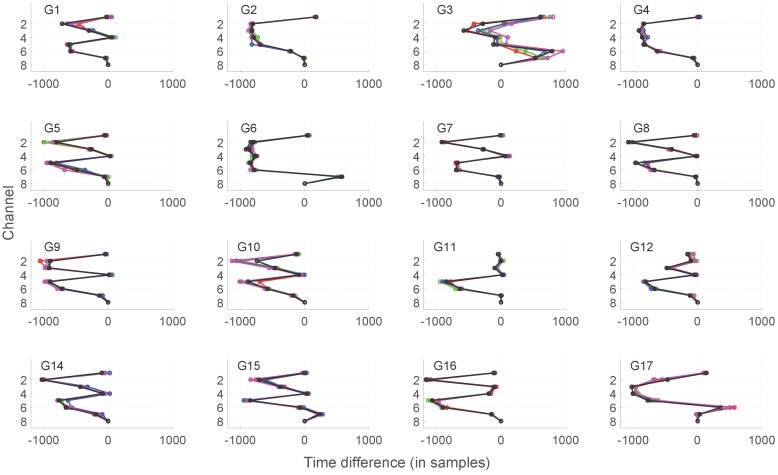
Activation profiles of eight muscles during golf swing for 16 different golf players. Note: channel 8 (left *trapezius*) was used as the reference for measuring time differences for remaining channels.

To get deeper insights into the variations of the muscle activation profiles, the profile representations $x=x_{1},...,x_{7}$ were visualized. Figure 7 presents the resulting map of the profiles. Activation profiles indicate that each individual player exhibits a rather stable activation pattern. Stable refers to most players having both stable activation sequences and time differences, except for players G3 and G10 (see Figure 7). Several players also exhibit almost identical activation patterns. Therefore, it is interesting to investigate the relation of the activation profile to parametrically- or biomechanically-effective transition. Nonetheless, profiles themselves can hardly be suitable as EMG features for prediction of player-specific effectiveness and exact club head speed or ball carry distance.

Interestingly, for golf player G15, correct biomechanically (see Appendix A), but not among the most effective players of the same sex, EMG activity in muscles related to the Channels 2 (right *EDC*), 3 (left *FCR*) and 5 (right *rhomboideus*) starts before the reference muscle (left *trapezius*) is activated, but EMG activity in muscle related to Channel 7 (left *rhomboideus*) starts after activation of the reference muscle (left *trapezius*).

Regarding the relation of muscle activation sequence to sex-specific effectiveness, the following insights were derived. Effective men (G5, G8 and G9) and women (G4 and G14) players had the reference muscle as lagging, and no channels were leading the reference; all channels were activated noticeably before the reference or simultaneously with it (hence, no activation times with high positive values). Profiles for effective men (G5, G8 and G9) had almost an identical shape, where Channels 1, 4, 8 were activated in a synchronized fashion and remaining channels in a specific order earlier. Profiles for effective women (G4 and G14) were distinct with regard to Channels 3–4, where player G14 with more scattered shots had Channel 4 synchronized to Channels 1 and 8 (as in effective men), and Channel 3 was activated a bit earlier; but similarly, effective player G4 had a rather unique profile shape with less synchronization to the reference channel. Most profiles (with the exception of G3, G6, G15 and G17 players), and especially effective men and women, revealed a consistent sequential order: avalanche effect, where Channels 5–7 formed a chain of activations with respect to the reference (Channel 8): first 5, then 6, then 7 and, finally, 8.

### 4.2. Predictive Performance

The total number of trees in RF was 5000. Several specific values of *q* ($\sqrt[{}]{p}$, $2\cdot \sqrt[{}]{p}$, *p*) in prediction using individual feature sets and all possible values of *q* (2–22) in decision-level fusion were tested and the best performing *q* setting retained.

#### 4.2.1. Detection Performance

**Figure 8:**
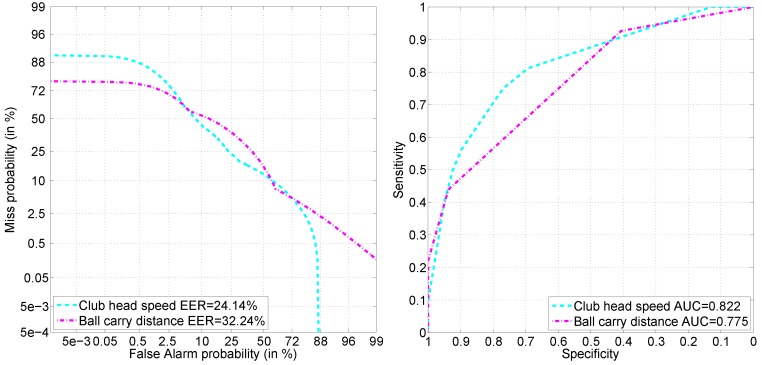
Decision-level fusion for detection task: evaluation by the detection error trade-off (DET) (**Left**) and ROC (**Right**) curves.

The detection task was more successful at predicting club head speed than ball carry distance, both when using individual feature sets (see Table 2) and when combining them through decision-level fusion (see Table 3 and Figure 8). The lowest EER using a single feature set was 32% for the speed and 39% for the distance (see Table 2).

The best individual feature sets were quite different with respect to the target attribute: the difference between correlations (*CorrDelta* and *StatsCorrDelta*) for speed and the difference between absolute concurrencies of peaks (*PeakAbsSyncDelta*), besides the absolute concurrency of the first peak (*PeakP1AbsSync*) for distance prediction.

**Figure 9:**
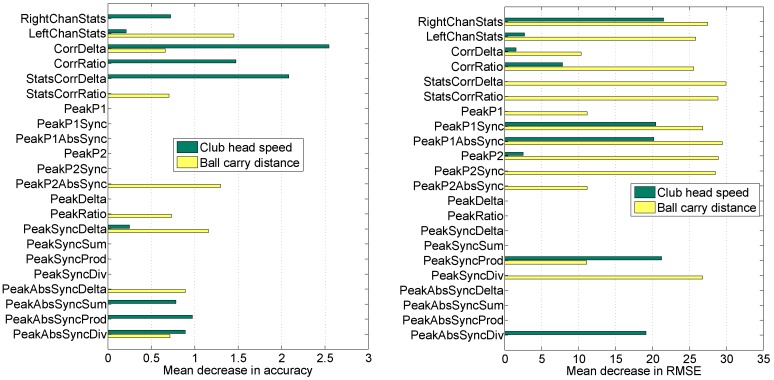
RF permutation-based variable importance in decision-level fusion for detection (**Left**) and regression (**Right**) tasks.

Decision-level fusion together with decision optimization achieved EER of 24.32% for the club head speed and 31.71% for the ball carry distance target attributes (see Table 3 and Figure 8). The decision optimization step reduced the need for various feature sets by more than half (see left side of Figure 9): ∼41% (nine out of 22) of meta-features were retained for speed and ∼36% (eight out of 22) of meta-features were retained for distance prediction.

Ranking of meta-features revealed similar tendencies as individual rankings of feature sets for speed, but not for distance. For example, the top three rankings for speed are identical (compare *Rank* of the club head speed in Table 2 with the left side of Figure 9 or see the detection part of Table B1 for convenience), but the top three rankings for distance are distinct (compare *Rank* of ball carry distance in Table 2 with the left side of Figure 9 or see the detection part of Table B2 for convenience). It appears that when combining feature sets through decision-level fusion, additional feature sets can become more important than individually best ones. For the distance case, such complimentary feature sets are the statistics of left channels (*LeftChanStats*) and the absolute concurrency of the second peak (*PeakP2AbsSync*).

#### 4.2.2. Regression Performance

**Figure 10:**
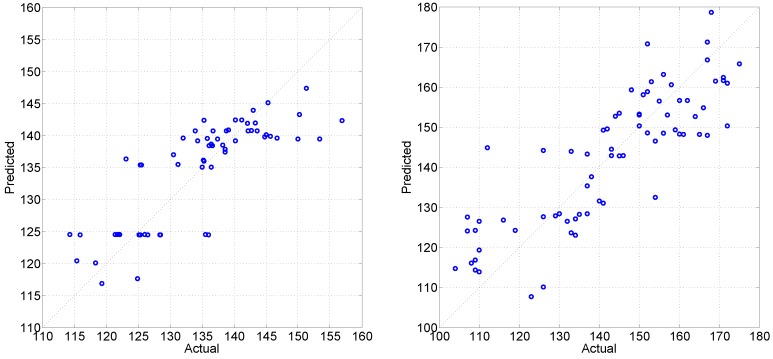
Decision-level fusion for regression task: results for club head speed (**Left**) and ball carry distance (**Right**) prediction. Note: the diagonal dotted line corresponds to an ideal prediction.

The regression models were more successful in predicting club head speed than ball carry distance, both when using individual feature sets (see Table 4) and when combining them (see Table 5 and Figure 10). The lowest MAPE using a single feature set was 4.7% for the speed and 8.7% for the distance (see Table 4). Such poor performance of individual feature sets for the regression task is unsatisfactory, since all performance metrics (RMSE, MAPE and correlation) appear to be worse than a naive guess that the effectiveness parameters of any swing in question simply are equal to the sex-specific average.

Best individual feature sets were quite different with respect to the target attribute: properties of the first peak (*PeakP1*), besides the difference between correlations (*StatsCorrDelta*) for the speed, and the ratio between absolute concurrencies of first and second peaks (*PeakSyncDiv*), besides statistics of left channels (*LeftChanStats*) for the distance prediction.

Decision-level fusion together with decision optimization proved to be successful in improving regression up to the satisfactory performance level and achieved MAPE of 3.22% for club head speed and 6.38% for ball carry distance target attributes (see Table 5). Nonetheless, the club head speed prediction (see the left side of Figure 10) defaults to the sex-specific average for women players, which could imply that exact club head speed prediction is not an easy task, either due to the task itself or due to the small sample of women participants. The decision optimization step reduced the need of various feature sets, but the reduction was more prominent for the speed than for the distance (see the right side of Figure 9): ∼41% (nine out of 22) meta-features retained for speed *versus*∼64% (14 out of 22) meta-features retained for distance prediction.

Ranking of the meta-features revealed no similar tendencies with individual rankings of feature sets, irrespective of the target attribute. For convenient comparison, see Appendix B (the regression part in Table B1 and Table B2). Here, we reiterate that when combining feature sets through decision-level fusion, additional feature sets can reveal themselves as more important than individually best ones. For the speed case, such complimentary feature sets are the statistics of the channels corresponding to the muscles on the right side of the body (*RightChanStats*) and multiplication of concurrencies for the first and the second peaks (*PeakSyncProd*). For the distance case, such complimentary feature sets are the difference between correlations (*StatsCorrDelta*) and the absolute concurrency of the first peak (*PeakP1AbsSync*).

#### 4.2.3. Summary of Performance

The top feature sets for the club head speed target attribute were statistics of activity in the muscles on the right side of the body (*RightChanStats*), difference between correlations (*StatsCorrDelta* and *CorrDelta*) and the ratio between absolute concurrencies of first and second peaks (*PeakAbsSyncDiv*). The top feature sets for the ball carry distance target attribute were the statistics of activity in the muscles on the left side of the body (*LeftChanStats*), absolute concurrency of the first peak (*PeakP1AbsSync*), properties of the second peak (*PeakP2*) and difference between absolute concurrencies of the first and second peaks (*PeakAbsSyncDelta*). Interestingly, even though muscle activity on both sides of the body is important, the importance of body side is target-related: the right side muscles are useful in predicting club head speed; meanwhile, the left side muscles are useful in predicting ball carry distance. For more details, see Appendix B.

In summary, channel statistics from both sides (*RightChanStats* and *LeftChanStats*) could be considered as the most informative features. The difference between Pearson and Spearman correlations for each channel pair with the statistical summary of these differences (*StatsCorrDelta*) is the next important feature set. Both peaks proved to be useful separately, but in rather different aspects: absolute concurrency of the first peak (*PeakP1AbsSync*) *versus* basic properties of the second peak (*PeakP2*). Feature sets combining position concurrency of both peaks, such as the ratio of absolute concurrencies (*PeakAbsSyncDiv*) or the difference between signed concurrencies (*PeakSyncDelta*), also appear to be valuable.

## 5. Discussion

The introduced muscle activation profiles helped to summarize an EMG activity onset sequence in the monitored channels during the golf swing and revealed that the profile pattern of each player is very stable. Moreover, effective players, especially among men, had almost identical profiles. For effective profiles, EMG activity onsets in right *FCR*, left *EDC* and left *trapezius* muscles happened almost simultaneously, whereas activity in right *EDC*, left *FCR*, right *rhomboideus* and right *trapezius* muscles was detected earlier and constituted a specific avalanche effect. The effect is defined as the following sequence of EMG activity onsets: right *rhomboideus*, then right *trapezius* and then left *rhomboideus*. Such a sequence is supported by previous studies [48,49], where right *rhomboideus* and right *trapezius* are reported as most active in the back swing. Furthermore, the delayed activation of the left *trapezius*, right *FCR* and left *ECR* muscles in the present study also conforms to previous findings, where high levels of activity in left *trapezius* [48] and forearm [49] muscles during forward swing were detected. Interestingly, we observed similar activation patterns of the right *FCR* and left *EDC* muscles among effective men and for one of the more effective women; however, the most effective woman had a rather unique activation pattern. Due to the small sample size, 10 men and five women, further research into sex-specific differences in forearm muscle activity is required. Increased knowledge on these differing and unique neuromuscular patterns may aid in the characterization of an effective movement pattern for the optimization of golf swing performance.

Developing and maintaining a golf swing characterized by biomechanically-correct transition is not an easy task. As can be seen in Appendix A, only one participant managed to sustain the correct order in all of his shots. This experimental data may indeed partially explain what many coaches experience and why they consider sequential movement of body segments a key technical parameter [5]. Nonetheless, even if correct biomechanically, the player was not among the most effective, possibly due to the lack of synchronization after the avalanche effect, which was revealed by activation sequence profiles.

Ranking of the feature sets found a difference in body side relevance with respect to the club head speed and ball carry distance target attributes. Results show that club head speed is better predicted from the statistics of the dynamics in the right side muscles, whereas ball carry distance is better predicted from the statistics of dynamics in the left side muscles.

Some EMG feature sets revealed their predictive power only when decisions from these feature sets were used in combination with decisions arising from other feature sets. Therefore, in Appendix B, rankings from various modeling aspects were estimated and averaged into several overall rankings, namely target-and-task-specific and target-specific ranks. Ultimate ranking, averaged over targets, revealed channel statistics from both body sides and correlation-related feature sets as the most informative. The peak-related feature sets were also valuable, especially for the ball carry distance target attribute, where of notable importance are the absolute concurrency of the first peak and the basic properties of the second peak. The ratio of absolute concurrencies and the difference between signed concurrencies of the peaks were useful for both target attributes.

## 6. Conclusions

The two highest peaks in the filtered EMG signal of each channel were used to extract swing segment, the part of EMG stream with the most pronounced muscle activity, potentially containing all swing phases, and calculate various feature sets. Peak-based modeling of EMG dynamics proved to be useful for predicting shot effectiveness. The detection of personal effectiveness was an easier task than regression, where an exact club head speed or ball carry distance had to be predicted. Despite the task, the individual feature sets were clearly outperformed by the decision-level fusion. Club head speed was more predictable than ball carry distance: the lowest EER in detection was 24.3% for speed and 31.7% for distance; the lowest MAPE in regression was 3.2% for speed and 6.4% for distance.

From the ranking of feature sets, we speculate that for right-handed golfers right side upper body muscle activity is directly related to club head speed, whereas left side upper body muscle activity could be related to adjustments of club face orientation directly before the moment of impact to achieve a successful long shot. EMG channel statistics from both body sides, correlations between channels and peak-related features (concurrency of the first peak, properties of the second peak, ratio of absolute concurrencies of the peaks and difference between signed concurrencies of the peaks) were among the most important predictors of golf shot effectiveness.

Muscle activation profiles helped to reveal individual sequences, which appeared to be stable from shot to shot, especially for the most effective players. EMG activity for effective profiles started with a common avalanche effect from a triad of muscles (right *rhomboideus*, right *trapezius*, left *rhomboideus*) followed by the simultaneous activation of another triad of muscles (right *FCR*, left *EDC* and left *trapezius*). The level of synchronicity in EMG activity onsets in right *FCR*, left *EDC* and left *trapezius* muscles at the moment of impact during golf swing appears to influence shot effectiveness more than the biomechanical correctness of swing kinematics.

Analysis of EMG data from more participants would be beneficial to strengthen the results and conclusions. Decision-level fusion of EMG features with biomechanical features, automatically extracted from video kinematics, could be an interesting future direction. For example, combining the prediction of club head speed with an estimated angle of ball trajectory could potentially improve the accuracy of ball carry distance prediction. Such contributions would expand the scope of wearable computing applications in golf, provide insights for golfers and instructors alike and help to improve carry distance drives.

## Figures and Tables

**Table 1:** Descriptions of feature sets. Note: the size count does not include sex.

#	Feature Set	Size	Short Description
*Channel statistics & correlations*			
1	RightChanStats	49	Duration of swing and statistics for the right-side channels
2	LeftChanStats	49	Duration of swing and statistics for the left-side channels
3	CorrDelta	28	Difference between Pearson and Spearman correlations
4	CorrRatio	28	Ratio of Pearson correlation to Spearman correlation
5	StatsCorrDelta	40	CorrDelta features and statistics of differences
6	StatsCorrRatio	40	CorrRatio features and statistics of ratios
*Properties of the 1st peak ($P_{1}$)*			
7	PeakP1	40	The main properties of the 1st peak
8	PeakP1Sync	40	Difference between positions and statistics
9	PeakP1AbsSync	40	Absolute difference between positions and statistics
*Properties of the 2nd peak ($P_{2}$)*			
10	PeakP2	40	The main properties of the 2nd peak
11	PeakP2Sync	40	Difference between positions and statistics
12	PeakP2AbsSync	40	Absolute difference between positions and statistics
*Properties of both peaks ($P_{1}$ & $P_{2}$)*			
13	PeakDelta	40	Difference between the main properties of peaks
14	PeakRatio	40	Ratio between the main properties of peaks
15	PeakSyncDelta	40	Differences between positions subtracted and statistics
16	PeakSyncSum	40	Differences between positions summed and statistics
17	PeakSyncProd	40	Differences between positions multiplied and statistics
18	PeakSyncDiv	40	Differences between positions divided and statistics
19	PeakAbsSyncDelta	40	Absolute differences between positions subtracted and statistics
20	PeakAbsSyncSum	40	Absolute differences between positions summed and statistics
21	PeakAbsSyncProd	40	Absolute differences between positions multiplied and statistics
22	PeakAbsSyncDiv	40	Absolute differences between positions divided and statistics

**Table 2:** RF OOB performance for the detection task.

#	Feature Set	Club Head Speed				Ball Carry Distance			
		C_llr_	EER, %	AUC	Rank	C_llr_	EER, %	AUC	Rank
1	RightChanStats	0.954	35.71	0.702	4	1.088	47.58	0.546	19
2	LeftChanStats	1.035	40.80	0.626	15	1.144	50.00	0.500	22
3	CorrDelta	0.914	32.04	0.742	1	1.093	49.00	0.515	20
4	CorrRatio	0.949	35.18	0.716	3	1.043	45.59	0.556	12
5	StatsCorrDelta	0.918	31.07	0.756	2	1.104	49.64	0.507	21
6	StatsCorrRatio	0.976	37.06	0.682	6	1.071	48.23	0.534	17
7	PeakP1	1.036	46.06	0.557	16	1.070	49.71	0.505	16
8	PeakP1Sync	1.086	48.19	0.535	22	1.004	40.64	0.620	5
9	PeakP1AbsSync	1.018	41.10	0.611	11	0.981	41.01	0.638	2
10	PeakP2	0.961	33.31	0.708	5	1.021	44.64	0.591	6
11	PeakP2Sync	0.991	41.17	0.637	7	1.063	43.43	0.589	15
12	PeakP2AbsSync	1.004	41.23	0.620	8	1.035	41.10	0.641	11
13	PeakDelta	1.040	45.07	0.559	17	0.997	36.99	0.650	3
14	PeakRatio	1.069	50.00	0.500	21	0.999	38.20	0.647	4
15	PeakSyncDelta	1.016	42.53	0.616	9	1.062	46.43	0.561	14
16	PeakSyncSum	1.053	44.50	0.577	19	1.033	42.59	0.609	9
17	PeakSyncProd	1.034	43.68	0.590	14	1.051	45.62	0.574	13
18	PeakSyncDiv	1.017	43.80	0.599	10	1.022	42.83	0.601	7
19	PeakAbsSyncDelta	1.050	47.82	0.535	18	0.975	39.05	0.661	1
20	PeakAbsSyncSum	1.029	41.95	0.617	13	1.030	43.21	0.591	8
21	PeakAbsSyncProd	1.019	37.80	0.648	12	1.033	45.19	0.587	10
22	PeakAbsSyncDiv	1.057	49.03	0.517	20	1.079	47.54	0.547	18

**Table 3:** Decision-level fusion for detection task: summary of results. Note: 95CI stands for 95% confidence interval (mean and corresponding 95% CI are obtained after repeating the task 100 times).

Target Attribute	C_llr_ ± 95CI	EER ± 95CI, %	AUC ± 95CI
Club head speed	0.832 ± 0.001	24.32 ± 0.18	0.825 ± 0.001
Ball carry distance	0.895 ± 0.000	31.71 ± 0.14	0.779 ± 0.002

**Table 4:** RF OOB performance for the regression task.

#	Feature Set	Club Head Speed				Ball Carry Distance			
		RMSE	MAPE, %	Correl.	Rank	RMSE	MAPE, %	Correl.	Rank
1	RightChanStats	8.39	5.07	0.580	7	16.95	10.41	0.519	15
2	LeftChanStats	8.43	5.17	0.575	10	14.72	8.99	0.670	2
3	CorrDelta	9.47	5.78	0.394	21	19.69	12.06	0.117	22
4	CorrRatio	9.78	5.88	0.315	22	19.52	11.99	0.172	21
5	StatsCorrDelta	7.85	4.82	0.648	2	16.69	10.08	0.539	11
6	StatsCorrRatio	8.11	4.96	0.616	4	17.01	10.18	0.513	16
7	PeakP1	7.73	4.70	0.661	1	16.43	10.38	0.559	9
8	PeakP1Sync	8.80	5.29	0.519	16	17.32	10.90	0.486	18
9	PeakP1AbsSync	8.68	5.47	0.539	14	16.06	9.75	0.586	6
10	PeakP2	8.67	5.28	0.540	13	15.56	9.30	0.619	4
11	PeakP2Sync	9.09	5.54	0.470	19	16.71	10.20	0.538	12
12	PeakP2AbsSync	9.12	5.41	0.466	20	17.91	10.94	0.428	20
13	PeakDelta	8.14	4.92	0.612	5	16.17	10.05	0.578	7
14	PeakRatio	8.08	4.98	0.621	3	16.19	9.95	0.577	8
15	PeakSyncDelta	8.25	4.85	0.599	6	15.91	10.09	0.596	5
16	PeakSyncSum	8.80	5.24	0.520	15	17.13	10.55	0.503	17
17	PeakSyncProd	8.93	5.33	0.499	17	16.91	10.41	0.522	14
18	PeakSyncDiv	8.42	5.02	0.576	9	14.29	8.72	0.693	1
19	PeakAbsSyncDelta	8.51	5.14	0.563	11	16.50	10.18	0.554	10
20	PeakAbsSyncSum	8.65	5.20	0.542	12	16.81	10.45	0.530	13
21	PeakAbsSyncProd	8.98	5.41	0.491	18	17.72	10.89	0.448	19
22	PeakAbsSyncDiv	8.41	5.05	0.578	8	15.47	9.60	0.625	3

**Table 5:** Decision-level fusion for regression task: summary of results. Note: 95CI stands for 95% confidence interval (mean and corresponding 95% CI are obtained after repeating the task 100 times).

Target Attribute	RMSE ± 95CI	MAPE ± 95CI, %	Correl. ± 95CI
Club head speed	5.70 ± 0.01	3.22 ± 0.01	0.813 ± 0.001
Ball carry distance	10.70 ± 0.04	6.38 ± 0.04	0.842 ± 0.001

**Figure A1:**
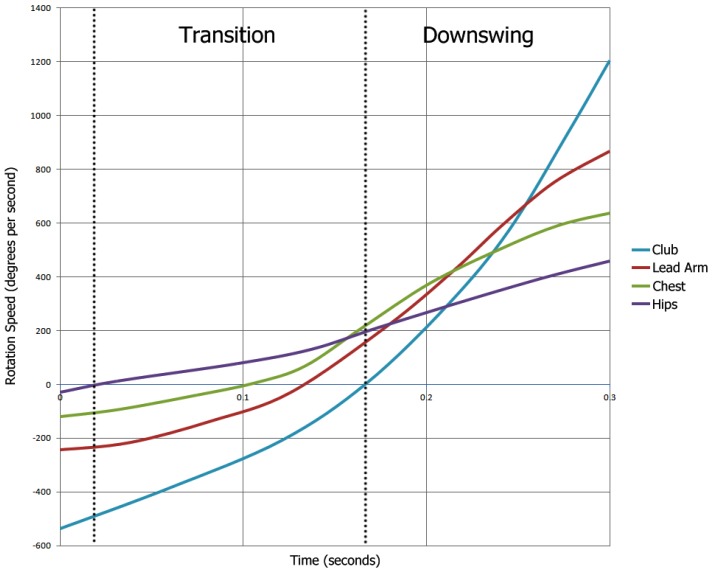
Illustration of a biomechanically-correct transition in a golf swing. The golf swing progresses in time on the *X* (horizontal) axis from left to right, and the speed of player’s body parts is on the *Y* (vertical) axis.

**Figure A2:**
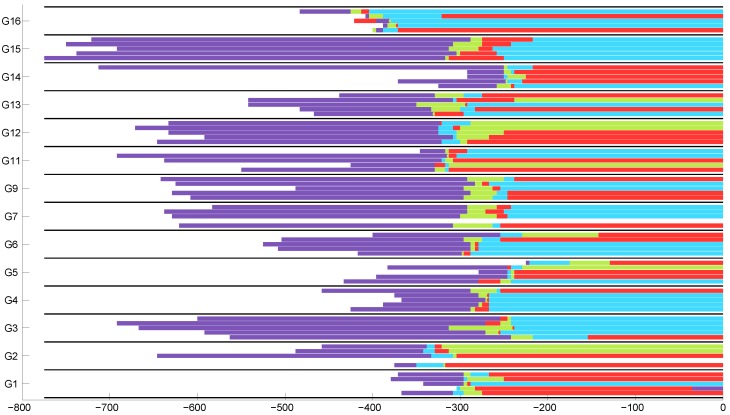
Illustration of transition sequences for 14 golf players (∼5 shots each), where color change means the change of rotation direction. The sequence of colors (violet, green, red and blue) for player G15 corresponds to the correct transition sequence (pelvis, thorax, arm and club). Note: position in samples on the *X* (horizontal) axis is shown, where 0 corresponds to the moment of impact.

## Appendix A.

Data on swing kinematics were collected using a four-sensor Polhemus Liberty electromagnetic motion capture system (Polhemus Inc., Colchester, VT, USA), operating at 120 Hz. Fifteen landmarks were digitized to define segment lengths, orientations and joint axes (see last column of Table A1). The swing events were determined from sensors on the club; the topmost position of back swing was determined as the moment the sensor on the club started to rotate with a positive direction around the global *Y* axis; and impact was determined as the moment at which a rapid deceleration of the club sensor in the down swing was noticed. Angular velocities and displacements of the pelvis, thorax and lead arm were calculated using standard biomechanical principles in conjunction with advanced motion measurement software (AMM 3D, Phoenix, AZ, USA).

In Figure A1, negative speeds reflect movement away from the ball, the back swing, while positive speeds indicate movement towards the ball, the down swing. The transition phase is defined as the interval between two points of rotation direction change, namely the direction of the pelvis and the club. The sequence of colors, violet, green, red and cyan, corresponds to the correct order of transitions, hips (pelvis), chest (thorax), arm and club.

**Table A1:** Sensor and landmark positions.

Body Part	Sensor Placement	Landmarks Used for Digitization
Club	Below grip	Top of grip
		Hozel
		Club head, bottom groove at heel
		Club head, bottom grove at toe
		Club head, top groove at toe
Left arm	Behind the elbow	Left acromion process
Thorax	On T5	Lateral epicondyle
		Medial epicondyle
Thorax/upper-body	On T5	Left acromion process
		Right acromion process
		Right side mid thorax, high
		Right side mid thorax, low
Pelvis	Sacrum	Left greater trochanter
		Right greater trochanter
		Point above left greater trochanter

Figure A2 presents experimental data illustrating transition sequences for fourteen experienced golf players. In Figure A2, color change means the change of rotation direction, and ’0’ on the *X* axis stands for the moment of impact. The swings of two players (G8 and G10) were not recorded, and the swings of one extra player (G13) were included instead; also, data for some shots (of G2 and G7) were missing. Only one player (G15) maintains the correct order in all five shots.

## Appendix B.

**Table B1:** Ranking of feature sets for club head speed prediction. Ind., individual; Imp., importance; Avg., average.

#	Feature Set	Ranking for Detection Task				Ranking for Regression Task				Overall Ranking	
		Ind.	Imp.	Avg.	Rank	Ind.	Imp.	Avg.	Rank	Avg.	Rank
1	RightChanStats	4	7	5.5	4	7	1	4.0	1	4.75	1
2	LeftChanStats	15	9	12.0	8	10	7	8.5	3	10.25	6
3	CorrDelta	1	1	1.0	1	21	9	15.0	15	8.00	3
4	CorrRatio	3	3	3.0	3	22	6	14.0	13	8.50	4
5	StatsCorrDelta	2	2	2.0	2	2	22	12.0	9	7.00	2
6	StatsCorrRatio	6	22	14.0	11	4	22	13.0	11	13.50	11
7	PeakP1	16	22	19.0	17	1	22	11.5	8	15.25	14
8	PeakP1Sync	22	22	22.0	22	16	3	9.5	5	15.75	15
9	PeakP1AbsSync	11	22	16.5	15	14	4	9.0	4	12.75	9
10	PeakP2	5	22	13.5	10	13	8	10.5	7	12.00	8
11	PeakP2Sync	7	22	14.5	12	19	22	20.5	21	17.50	19
12	PeakP2AbsSync	8	22	15.0	13	20	22	21.0	22	18.00	20
13	PeakDelta	17	22	19.5	18	5	22	13.5	12	16.50	17
14	PeakRatio	21	22	21.5	21	3	22	12.5	10	17.00	18
15	PeakSyncDelta	9	8	8.5	6	6	22	14.0	13	11.25	7
16	PeakSyncSum	19	22	20.5	20	15	22	18.5	19	19.50	22
17	PeakSyncProd	14	22	18.0	16	17	2	9.5	5	13.75	12
18	PeakSyncDiv	10	22	16.0	14	9	22	15.5	16	15.75	15
19	PeakAbsSyncDelta	18	22	20.0	19	11	22	16.5	17	18.25	21
20	PeakAbsSyncSum	13	6	9.5	7	12	22	17.0	18	13.25	10
21	PeakAbsSyncProd	12	4	8.0	5	18	22	20.0	20	14.00	13
22	PeakAbsSyncDiv	20	5	12.5	9	8	5	6.5	2	9.50	5

Ranking based on the performance of the individual feature sets (denoted as *Ind.* in Table B1 and Table B2) was averaged with ranking based on variable importance of the meta-learner RF (denoted as *Imp.* in Table B1 and Table B2) to reveal the final ranking (denoted as *Rank* in Table B1 and Table B2). Obtained task-specific rank averages (denoted as *Avg.* in Table B1 and Table B2) were further averaged to reveal target-specific overall ranking for club head speed (Table B1) and ball carry distance (Table B2).

**Table B2:** Ranking of feature sets for ball carry distance prediction.

#	Feature Set	Ranking for Detection Task				Ranking for Regression Task				Overall Ranking	
		Ind.	Imp.	Avg.	Rank	Ind.	Imp.	Avg.	Rank	Avg.	Rank
1	RightChanStats	19	22	20.5	21	15	6	10.5	8	15.50	16
2	LeftChanStats	22	1	11.5	5	2	9	5.5	4	8.50	2
3	CorrDelta	20	8	14.0	12	22	14	18.0	20	16.00	18
4	CorrRatio	12	22	17.0	17	21	10	15.5	16	16.25	19
5	StatsCorrDelta	21	22	21.5	22	11	1	6.0	5	13.75	14
6	StatsCorrRatio	17	7	12.0	6	16	4	10.0	7	11.00	7
7	PeakP1	16	22	19.0	20	9	12	10.5	8	14.75	15
8	PeakP1Sync	5	20	12.5	9	18	7	12.5	10	12.50	11
9	PeakP1AbsSync	2	22	12.0	6	6	2	4.0	2	8.00	1
10	PeakP2	6	21	13.5	11	4	3	3.5	1	8.50	2
11	PeakP2Sync	15	22	18.5	19	12	5	8.5	6	13.50	12
12	PeakP2AbsSync	11	2	6.5	3	20	11	15.5	16	11.00	7
13	PeakDelta	3	22	12.5	9	7	22	14.5	14	13.50	12
14	PeakRatio	4	5	4.5	2	8	22	15.0	15	9.75	6
15	PeakSyncDelta	14	3	8.5	4	5	22	13.5	12	11.00	7
16	PeakSyncSum	9	22	15.5	15	17	22	19.5	21	17.50	21
17	PeakSyncProd	13	22	17.5	18	14	13	13.5	12	15.50	16
18	PeakSyncDiv	7	22	14.5	13	1	8	4.5	3	9.50	5
19	PeakAbsSyncDelta	1	4	2.5	1	10	22	16.0	18	9.25	4
20	PeakAbsSyncSum	8	22	15.0	14	13	22	17.5	19	16.25	19
21	PeakAbsSyncProd	10	22	16.0	16	19	22	20.5	22	18.25	22
22	PeakAbsSyncDiv	18	6	12.0	6	3	22	12.5	10	12.25	10

**Table B3:** Ultimate ranking of feature sets for the prediction of golf shot effectiveness.

#	Feature Set	Club	Dist	Avg.	Rank
1	RightChanStats	4.75	15.50	10.13	2
2	LeftChanStats	10.25	8.50	9.38	1
3	CorrDelta	8.00	16.00	12.00	8
4	CorrRatio	8.50	16.25	12.38	10
5	StatsCorrDelta	7.00	13.75	10.38	4
6	StatsCorrRatio	13.50	11.00	12.25	9
7	PeakP1	15.25	14.75	15.00	18
8	PeakP1Sync	15.75	12.50	14.13	14
9	PeakP1AbsSync	12.75	8.00	10.38	4
10	PeakP2	12.00	8.50	10.25	3
11	PeakP2Sync	17.50	13.50	15.50	20
12	PeakP2AbsSync	18.00	11.00	14.50	15
13	PeakDelta	16.50	13.50	15.00	18
14	PeakRatio	17.00	9.75	13.38	12
15	PeakSyncDelta	11.25	11.00	11.13	7
16	PeakSyncSum	19.50	17.50	18.50	22
17	PeakSyncProd	13.75	15.50	14.63	16
18	PeakSyncDiv	15.75	9.50	12.63	11
19	PeakAbsSyncDelta	18.25	9.25	13.75	13
20	PeakAbsSyncSum	13.25	16.25	14.75	17
21	PeakAbsSyncProd	14.00	18.25	16.13	21
22	PeakAbsSyncDiv	9.50	12.25	10.88	6

Ultimate ranking (denoted as *Rank* in Table B3) of feature sets is estimated after combining the target-specific overall rank averages (replicated in the *Club* and *Dist* columns of Table B3) into the ultimate average (denoted as *Avg.* in Table B3).
